# A Typical Bilateral Atrial Myxoma: A Case Report

**DOI:** 10.1155/2012/460268

**Published:** 2012-07-12

**Authors:** Zhenghua Xiao, Wei Meng, Da Zhu, Eryong Zhang

**Affiliations:** Department of Thoracic and Cardiovascular Surgery, West China Hospital, Sichuan University, Sichuan, Chengdu 610041, China

## Abstract

Myxoma is a rare type of tumor which have an incidence of 0.0017% among the general population. Cardiac myxomas which arise from two different heart chambers is even extremely rare; we herein report a unique case of male patient with bilateral myxoma.

## 1. Introduction

Primary cardiac tumor is an extremely rare type of tumor, accounting for less than 0.2% of all tumors found in human being. Unlike other types of neoplastic disease, approximately 75% of intracardiac is biological benign and often asymptomatic. But this type of disease is still associated with several major complications including thrombus, valve obstruction, or even sudden cardiac death due to its unique location.

 Myxoma is the most common intracardiac tumor, which has an incidence of 0.0017% in the general population. It is commonly located in the left atrium and mainly originate from an area in the atrial septum near the fossa ovalis [1, 2]. Right atrial (RA) myxomas are less common. Also reported in the literature, it could be found in the aorta, pulmonary artery, ventricles, vena cava, or even other organs [3].

 Cardiac myxoma that arise from two different heart chambers are extremely rare; we herein report a unique case of male patient with biatrial myxoma.

## 2. Case Presentation

**Figure 1:**
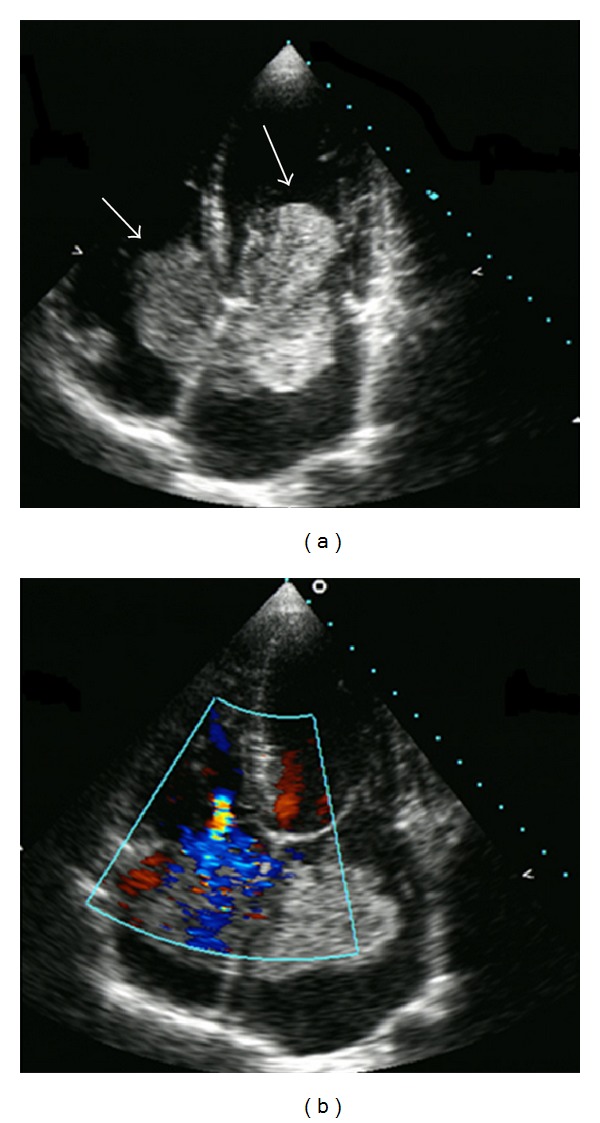
Echocardiogram showing bilateral atrial tumors (arrows), large left atrial mass protruding through the mitral valve during diastole. There was a mild tricuspid regurgitation on the color Doppler examination.

A 39-year-old male patient was referred to our hospital due to dyspnea and palpitation which began recently and became increasingly worse. Under cardiac auscultation, a 3/6 systolic murmur without a tumor plop was heard. Electrocardiogram showed sinus rhythm. Transthoracic echocardiogram revealed two intracardiac masses which attached to the atrial septum, with a larger one in the LA (69 mm × 39 mm) while a relatively small one in RA, both of these masses were prolapsing through the mitral/tricuspid valve during diastole (Figure 1). Doppler ultrasound confirms both transmitral and tricuspid flow acceleration. In order to rule out other secondary or metastatic tumors, a chest radiography showed clear lung field and the CT of abdomen ruled out any intra-abdominal pathology or source of cardiac metastasis.

**Figure 2:**
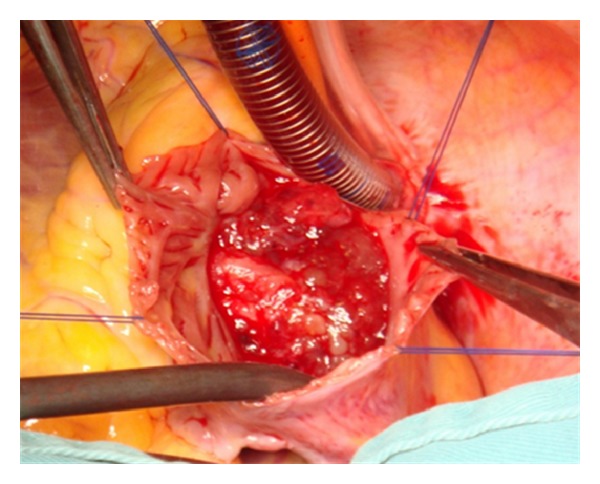
Opening the right atrium with myxoma attached to the interatrial septum (picture is taken from the head end of the patient).

After providing written informed consent, the patient underwent masses resected surgery under the cardiopulmonary bypass. During the operation, an incision was made in the right atrium exposing a 30 by 40 mm gelatinous mass, which was attached to the interatrial septum (Figure 2). The second gelatinous mass was found occupying a great part of the LA. The base of the second mass was attached to the left side of interatrial septum immediately opposite to the attachment of the RA septum. 

**Figure 3:**
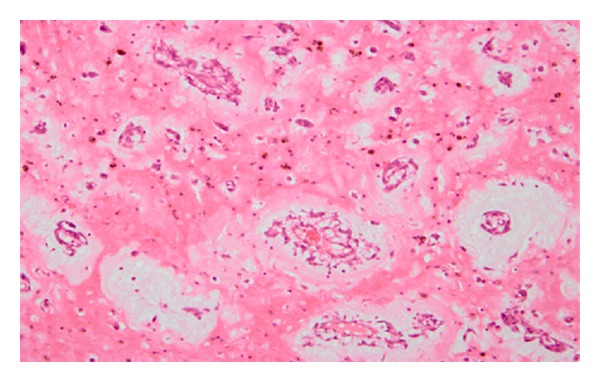
Microscopic features of the specimen: it composed of satellite myxoma cells, inflammatory cells, and much basophilic substance (200×).

Both masses were resected carefully to avoid manipulation and fragmentation of the tumor. The left-sided mass was large and involved part of the septum, so we also removed the part of the septum. Then a bovine pericardial patch was used to reconstruct the atrial septum. Right and left ventricles were visually inspected through their valves and no other masses were found. Meanwhile, the resected mass was sent for histological assessment and revealed the histopathological features of benign cardiac myxoma. It was composed of satellite myxoma cells, inflammatory cells, and much basophilic substance (Figure 3). 

The echocardiogram, done during immediate postoperative period and prior to the discharge, showed no residual myxoma and mitral/tricuspid regurgitation.

## 3. Discussion

 Myxoma is the most prevalent primary cardiac tumor, which is commonly located in the left atrium. Right atrial myxomas are uncommon, being three to four times less frequent than those located in the LA [3]. Multiple intracardiac myxoma accounting for less than 5% among all the cases, and the biatrial myxoma is fewer than 2.5% [4].

 Early diagnosis for intracardiac tumor is difficult because the symptoms are frequently nonspecific [5]. Symptoms for this type of patient are often due to endomyocardial flow obstruction or peripheral embolization. It could also be intermittent, resulting from occasional prolapse of pedunculated and mobile tumors through the atrioventricular valves into the ventricle [6]. Valvular obstruction may lead to dyspnoea, arrhythmias, precordial uneasiness, dizziness and syncopal episodes, heart failure, and acute pulmonary edema. Patients with myxoma may also present with Raynaud phenomenon, anemia, fever, weight loss, hypergammaglobulinemia, and an increased erythrocyte sedimentation rate due to the interleukin-6 release from the tumour cells [7]. 

The heart auscultation can be quite similar to that of mitral valve disease, and may be associated with a tumoral sound. In this patient, we can hear a grade 3/6 ejection systolic murmur without a tumor plop. The most useful examination in the diagnosis is the echocardiogram which could confirm the location and extention of myxomas with a highly diagnostic sensitivity. Plain chest X-ray or CT scan is not diagnostic except for the pulmonary metastasis in cases of malignancy or the metastatic tumor [8].

Although histopathologically benign, myxomas recurrences and malignant forms have been also described. The recurrence, rate is estimated as 5% and usually this happens up to 5 years postoperatively. It is believed that the risk of tumour recurrence is higher in younger patients, in familial forms of myxoma (hence the importance of detailed family history), and in multilocular myxomas [9]. Tumour recurrence is possible in cases of incomplete resection, implantation of tumour cells during tumour excision, and regrowth in another location. In order to avoid this, open heart chambers were examined carefully and the manipulations during the procedure minimized, to prevent intraoperative embolization until aortic cross-clamping. Wide resection of the myxoma should be done including adjacent cardiac tissue and septum if required. In our case the surgical approach was biatrial through the right and left atrium, we removed myxomas and a part of adjacent septum at the same time, a bovine pericardial patch was used to reconstruct the atrial septum. It would be necessary to avoid tumor regrowth. 

Biatrial myxoma is an extremely rare occurrence, Once a diagnosis is established, these tumors should be removed to prevent further cardiac or neurologic sequelae. With complete resection the recurrence rate should be less than 5%, and patients should be followed up with serial echocardiography.
